# EVL Promotes Osteo-/Odontogenic Differentiation of Dental Pulp Stem Cells via Activating JNK Signaling Pathway

**DOI:** 10.1155/2023/7585111

**Published:** 2023-01-12

**Authors:** Kangrui Zeng, Qiongyi Kang, Yutong Li, Weiping Li, Qing Cheng, Wenwei Xia

**Affiliations:** ^1^Department of Endodontics and Operative Dentistry, Shanghai Ninth People's Hospital, Shanghai Jiao Tong University School of Medicine, College of Stomatology, Shanghai Jiao Tong University, National Center for Stomatology, National Clinical Research Center for Oral Diseases, Shanghai Key Laboratory of Stomatology, Shanghai, China; ^2^Department of stomatology, The Affiliated Jiangyin People's Hospital of Southeast University Medical College, Jiangyin, Jiangsu, China

## Abstract

**Objective:**

Human dental pulp stem cells (hDPSCs) were recognized as a suitable and promising source of stem cells in dental pulp regeneration. However, the mechanism by which hDPSCs differentiation into osteo-/odontogenic lineage remains unclear. Ena/VASP-like protein (EVL) has been found to be involved in diverse biological processes. In this study, we explored the role and underlying mechanism of EVL in osteo-/odontogenic differentiation of hDPSCs.

**Methods:**

Expression of EVL was detected in hDPSCs by quantitative reverse transcription polymerase chain reaction (qRT-PCR) and western blot (WB) analyses during osteo-/odontogenic differentiation. The function of EVL in osteo-/odontogenic differentiation and involvement of MAPK signaling pathways were evaluated by alkaline phosphatase (ALP) staining and activity, alizarin red staining (ARS), and qRT-PCR and western blot analyses.

**Results:**

The expression of EVL was upregulated during osteo-/odontogenic differentiation of hDPSCs. Overexpression of EVL significantly increased osteo-/odontogenic capacity of hDPSCs, which was reflected in increased alkaline phosphatase (ALP) staining, ALP activity, mineralized nodule formation, and the expressions of genes related to osteo-/odontogenic differentiation, while downregulation of EVL inhibited it. In addition, EVL activated the JNK pathway and phosphorylation of p38 MAPK during differentiation procedure of hDPSCs. The EVL-enhanced differentiation of DPSCs was suppressed by blocking the JNK pathway, rather than the p38 MAPK pathway.

**Conclusion:**

EVL promotes the osteo-/odontogenic differentiation of hDPSCs by activating the JNK pathway, providing a future target for osteo-/odontogenic differentiation and dental pulp regeneration.

## 1. Introduction

Pulpitis and periapical periodontitis have become common and widespread morbidities, endangering people's general and mental health [1, 2]. Nowadays the main clinical treatment is root canal therapy (RCT) which involves the extirpation of the entire dental pulp and filling with an artificial material, resulting in a higher incidence of extraction. However, teeth after RCT are susceptible to fracture and more prone to mechanical damage, resulting in a higher incidence of extraction [3–5]. Recently, mesenchymal stem cell- (MSC-) based tissue engineering has shown immense potential for dental pulp regeneration, which was proposed as an alternative to conventional endodontic treatment [6, 7]. Dental pulp stem cells (hDPSCs) are a type of odontogenic MSCs with self-renewal capacity and multiple differentiation potentials, initially isolated in 2000 by Gronthos et al. [8–11]. Compared with other MSCs, hDPSCs exhibit greater clonality, proliferation and mineralization potential [12, 13], and can be obtained via simple, minimally invasive procedures [14]. Consequently, hDPSCs are regarded as a promising candidate for dental pulp regeneration [6, 15]. Yet, the osteo-/odontogenic differentiation of hDPSCs is a complex process associated with various factors, which have not been fully explored. Therefore, understanding the functions of hDPSCs during differentiation and the underlying molecular mechanisms is of great importance.

Actin cytoskeleton plays an important role in various physiological processes, such as cell proliferation, cell adhesion, cell motility, and signal transduction [16–19]. Recently, increasing attention has been paid to the relationship between actin cytoskeleton and stem cell differentiation [20–22]. It has been shown that the actin cytoskeleton is involved in regulating osteo-/odontogenic differentiation [23–25]. Inhibition of actin-depolymerizing factors, such as Cofilin1 and Destrin, or treatment with filament-stabilizing drug Jasplakinolide (JAS) could facilitate osteogenesis differentiation of MSCs [26, 27]; likewise, cytochalasin D treatment can inhibit MSC osteogenesis differentiation by decreasing actin polymerization [28]. Inhibition of actin-related proteins-2/3 (Arp2/3) complex recruited to actin filaments to develop a branched actin network could entirely prevent osteogenesis, causing adipogenic differentiation [29]. It has been noted that cytoskeleton actin filament dynamics and organization are regulated by various actin-binding proteins (ABPs) [27, 30, 31], such as Arp2/3 complex, filamin A, vinculin, cofilin, and Ena/VASP family proteins, indicating a significant role of ABPs in modulating the differentiation of hDPSCs.

Ena/VASP-like (EVL), a member of the Ena/VASP protein family, is implicated as an actin assembly factor that facilitates the formation of interdigitated actin microspikes mediated by Arp2/3 and CRMP-1 [32, 33]. Additionally, EVL regulates the cytoskeleton remodeling by nucleating, polymerizing, and elongating actins [34–36]. A recent study has shown that EVL played a crucial role in regulating hematopoietic differentiation [37]. However, the function of EVL in the differentiation process of hDPSCs has yet not been investigated.

In this study, we confirmed the vital role of EVL in modulating osteo-/odontogenic differentiation of hDPSCs, and clarified the involvement of JNK signaling pathway in EVL-induced osteo-/odontogenic differentiation, providing a better understanding of the molecular mechanisms that drove hDPSCs differentiation.

## 2. Materials and Methods

### 2.1. Cell Isolation and Culture

All teeth tissues were collected at the Ninth People's Hospital affiliated with Shanghai Jiao Tong University School of Medicine. The experimental procedures were approved by the Medical Ethics Committee of the Ninth People's Hospital (SH9H-2021-A273-SB). The dental pulp tissue was collected and extracted from third molars and premolars of healthy patients aged between 18 and 25 years old. Briefly, the dental pulp was washed with phosphate-buffered saline (PBS; pH 7.4) and digested in a mixed solution containing 3 mg/mL type I collagenase (Sigma-Aldrich, St. Louis, MO, USA) and 4 mg/mL dispase II (Sigma-Aldrich, St. Louis, MO, USA) for 30 minutes at 37°C. The isolated cells were cultured in the growth medium (GM), the high-glucose DMEM (HyClone, South Logan, UT, USA) supplemented with 10% fetal bovine serum, 100 U/mL penicillin, and 100 mg/mL streptomycin. All hDPSCs passaged 3-5 times were used for subsequent experiments.

### 2.2. Flow Cytometry

For phenotype identification, hDPSCs were rinsed with PBS and incubated with the primary antibodies including CD29-PE, CD31-FITC, CD34-FITC, CD44-FITC, CD45-FITC, and CD105-PE (BD Biosciences, CA, USA) and kept in dark at room temperature for 20 minutes. Isotype controls conjugated to FITC and PE were used as negative controls. The samples were immediately subjected to flow cytometric analysis on a FACSCalibur flow cytometer (Becton Dickinson).

### 2.3. Trilineage Differentiation Potential Assay

For osteogenesis, adipogenesis, and chondrogenesis analyses, cells were cultured in GM containing the high-glucose DMEM and 10% fetal bovine serum. After reaching 80% confluence, the medium was replaced with the osteogenesis-inducing medium (OM), the high-glucose DMEM supplemented with 10 mM *β*-glycerol phosphate, 0.01 *μ*M dexamethasone and 50 *μ*M ascorbic acid (Sigma-Aldrich, St. Louis, MO, USA). Then, cells were cultured for 21 days. Chondrogenic and adipogenic differentiation were induced by adipogenesis-inducing medium and chondrogenesis-inducing medium (Cyagen, Guangzhou, China). Alizarin red S staining (ARS), oil red O staining, and alcian blue staining were performed to evaluate osteogenic, adipogenic, and chondrogenic differentiation potentials.

### 2.4. Quantitative Real-Time PCR

Total RNA was extracted with TRIzol reagent (Invitrogen, Carlsbad, CA, USA) and quantified using a NanoDrop spectrophotometer (NanoDrop, Wilmington, DE). A PrimeScript reverse transcription kit (TAKARA, Shiga, Japan) was used to perform the reverse-transcriptional reactions, and genes were quantified with a LightCycler® 480 PCR system (Roche, Basel, Switzerland) using a SYBR Premix Ex Taq kits (TAKARA, Shiga, Japan). Glyceraldehyde 3-phosphate dehydrogenase (GAPDH) was used as an internal control. All primers were synthesized by Sangon Biotech (Shanghai, China). Primer sequences are listed in Table 1.

### 2.5. Western Blot

Cells were lysed in RIPA lysis buffer (Beyotime, Shanghai, China). The concentration of each protein sample was measured using a BCA assay kit (Sigma-Aldrich, St. Louis, MO, USA). The samples were separated by SDS-PAGE electrophoresis and transferred onto a PVDF membrane (Millipore, Billerica, MA, USA). The membrane was blocked with 5% dehydrated milk for 2 hours and then incubated with the primary antibodies at 4°C overnight. The information of primary antibodies used was as follows: RUNX2 (#12556, Cell Signaling Technology), EVL(#12536, Cell Signaling Technology), mitogen-activated protein kinase (MAPK) Family Antibody Sampler Kit (#9926, Cell Signaling Technology), Phospho-MAPK Family Antibody Sampler Kit (#9910, Cell Signaling Technology), DMP1 (PA5-88069, Invitrogen), DSPP (sc-73632, Santa Cruz), OSX (PA5-115697, Invitrogen), OCN (ab93876, Abcam), and GAPDH (#2118, Cell Signaling Technology). Proteins were quantified using the enhanced chemiluminescence kit (Pierce, IL, USA).

### 2.6. Alkaline Phosphatase (ALP) Staining and Activity Measurement

For ALP staining, cells were cultured in GM or OM for 3 and 7 days, then fixed in 4% paraformaldehyde and stained with a BCIP/NBT ALP Color Development Kit (Beyotime, Shanghai, China). The images were captured under an inverted phase contrast microscope.

An ALP Assay Kit (Beyotime, Shanghai, China) was used to analyze the ALP activity according to the manufacturer's instructions. ALP activity was detected by an ultraviolet spectrometer at 405 nm wavelength and the protein concentrations were measured using BCA assay kit. Relative ALP activity normalized to corresponding total protein contents was presented as units/mg protein.

### 2.7. Alizarin Red S (ARS) Staining and Quantitative Analysis

For ARS staining, cells were cultured in GM and OM for 21 days, fixed in 4% paraformaldehyde, and stained with 1% ARS (pH 4.3) for 15 min. The images indicating calcium deposition were captured under an inverted phase contrast microscope. For quantitative analysis of ARS, 10% cetylpyridinium chloride monohydrate (Sigma-Aldrich, St. Louis, MO, USA) was used to dissolve the calcium deposition. The absorbance was measured at 562 nm.

### 2.8. Cell Transfection

Specific siRNAs specifically targeting EVL (si-EVL) and the negative control (si-NC) were designed and synthesized by RiboBio (Guangzhou, China). For EVL overexpression experiment, the EVL gene was subcloned into the pEZ-M98 vector (GeneCopoeia, Inc.) to generate the pM-EVL plasmid. Cells were cultured and transfected with pM-EVL plasmid, scrambled negative control empty pEZ-M98 vector (pMV), si-EVL, and si-NC using Lipofectamine 3000 (Invitrogen, Carlsbad, CA, USA) according to the manufacturer's instructions. After 4 hours of transfection, the medium was replaced with the OM. The target sequences of the siRNAs are listed in Table 2.

### 2.9. Statistical Analysis

All quantitative results were presented as mean ± standard deviation (SD). Student's *t*-test and one-way analysis of variance (ANOVA) were performed to determine the statistical significance. All statistical calculations were conducted with SPSS13.0 (IBM SPSS Inc., Armonk, NY, USA). A *P* value ≤ 0.05 was considered statistically significant.

## 3. Results

### 3.1. Phenotype Identification and Trilineage Differentiation Potential of hDPSCs

**Figure 1:**
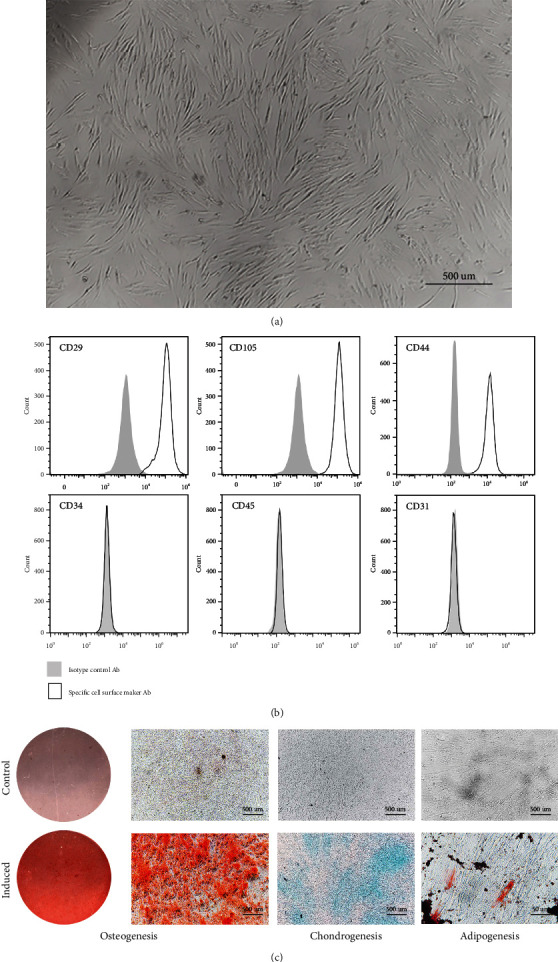
Isolation and identification of hDPSCs. (a) Morphology of hDPSCs. (b) Surface antigens of hDPSCs were assessed by flow cytometry. (c) Osteogenic capacity was confirmed with positive alizarin red staining. Adipogenic capacity was verified with positive oil red O staining. Chondrogenic capacity was determined by alcian blue staining. Scale bar = 500 *μ*M.

To identify the characteristics of hDPSCs, the morphology, immunophenotype, and multilineage differentiation potential analyses were performed. The morphology of primary hDPSCs presented a fibroblast- or spindle-like shape (Figure 1(a)). Flow cytometric analysis indicated that hDPSCs were stained positive for MSCs markers CD29, CD44, and CD105, but negative for leucocyte cell marker CD45 and platelet endothelial cell marker CD31 and CD34 (Figure 1(b)). Next, we performed a trilineage differentiation assay to confirm the pluripotency of hDPSCs. Extracellular calcium deposition was evaluated by ARS staining after 21 days of osteogenic induction. Evaluation of intracellular lipid accumulation was detected by oil red O staining after 21 days of adipogenic induction. Alcian blue staining exhibited positive glycosaminoglycan signal after 21 days (Figure 1(c)). All these data confirmed that hDPSCs were successfully isolated from the dental pulp.

### 3.2. Upregulation of EVL during Osteo-/Odontogenic Differentiation of hDPSCs

**Figure 2:**
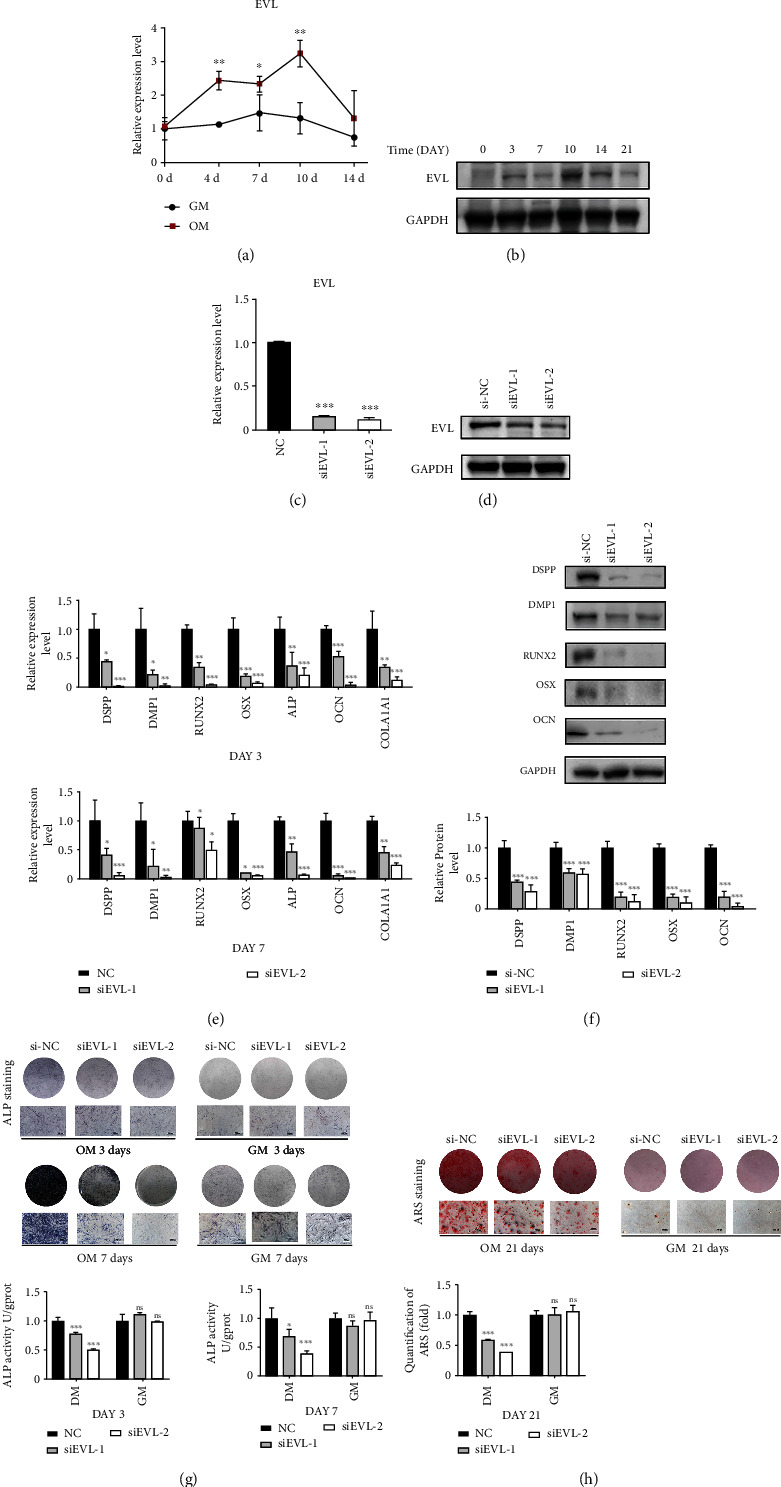
Knockdown of EVL inhibited the osteo-/odontogenic differentiation of hDPSCs. (a, b) The expression of EVL during osteo-/odontogenic differentiation was confirmed with qRT-PCR and western blot. (c, d) The knockdown efficiencies of siRNAs against EVL were verified by western blot assay and qRT-PCR. (e) The expressions of DSPP, DMP1, RUNX2, OCN, and OSX were analyzed in hDPSCs transfected with si-EVL and si-NC by western blot on the 7th day. (f) The expressions of DSPP, DMP1, ALP, RUNX2, OSX, OCN, and COL1A1 were analyzed in hDPSCs transfected with si-EVL and si-NC by qRT-PCR on the 3rd and 7th day. (g) ALP staining and quantitative intracellular ALP activity were measured in hDPSCs transfected with si-EVL and si-NC and cultured in growth medium (GM) or osteogenesis-inducing medium (OM) on the 3rd and 7th day. (h) Extracellular calcium deposition was visualized by alizarin red staining on the 21st day. Released dye in alizarin red staining was quantified by measuring absorbance at 562 nm. GAPDH was used as an internal control in qRT-PCR and western blot analyses. Data are shown as the mean ± SD (*n* = 3); ^∗^*P* ≤ 0.05, ^∗∗^*P* ≤ 0.01, ^∗∗∗^*P* ≤ 0.001.

To examine the correlation between EVL and osteo-/odontogenic differentiation of hDPSCs, we compared the expression level of EVL in hDPSCs cultured in OM versus GM. qRT-PCR assay revealed that the expression of EVL was significantly elevated in the osteogenesis-inducing group compared with the control group (Figure 2(a)). Furthermore, western blot analysis showed that the expression of EVL were found to be significantly increased since day 0 until day 10 while a decreasing trend was observed since day 14 until day 21 (Figure 2(b)).

### 3.3. Knockdown of EVL-Impaired Osteo-/Odontogenic Differentiation of hDPSCs

To evaluate the function of EVL in hDPSCs, we silenced EVL expression using siRNAs (si-EVL). Two siRNAs targeting distinct regions of EVL were designed to exclude possible off-target effects. The EVL knockdown efficiency was confirmed using qRT-PCR and western blot analyses (Figures 2(c) and 2(d)). Then, we explored whether EVL silencing regulated the osteo-/odontogenic capacity of hDPSCs. We examined the impact of EVL on key osteo-/odontogenic markers in hDPSCs. After osteogenic induction for 3 and 7 days, the silencing of EVL gene resulted in a significant decrease in DSPP, DMP1, ALP, RUNX2, OSX, OCN, and COL1A1 mRNA expression levels (Figure 2(e)). The protein levels of DSPP, DMP1, RUNX2, OSX, and OCN were distinctively downregulated after osteogenic induction for 7 days (Figure 2(f)). Furthermore, ALP activity assay and ARS staining for extracellular mineralization nodules were employed to analyze early and late stages of osteo-/odontogenic differentiation of hDPSCs. EVL silencing inhibited osteo-/odontogenic differentiation in OM-cultured hDPSCs, as indicated by lower ALP staining and ALP activity (Figure 2(g)), and fewer mineralization nodules (Figure 2(h)), but no difference was observed for GM-cultured hDPSCs.

### 3.4. EVL Overexpression Promoted Osteo-/Odontogenic Differentiation of hDPSCs

**Figure 3:**
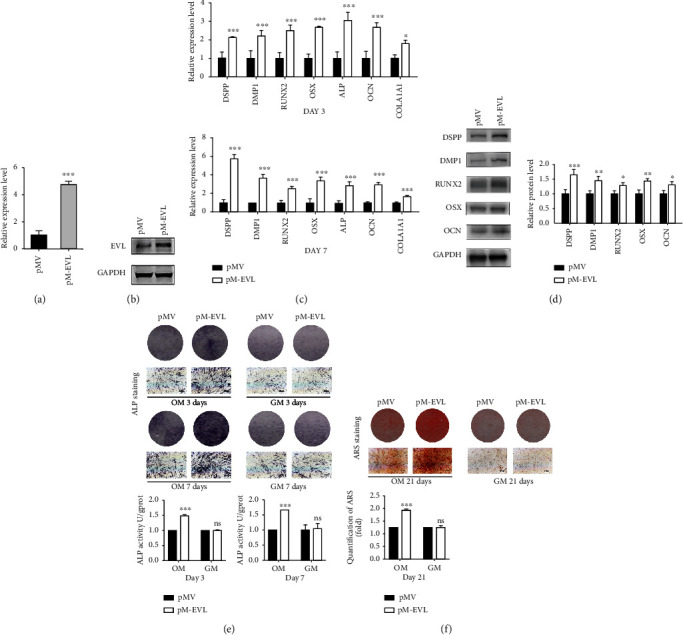
Overexpression of EVL promoted the osteo-/odontogenic differentiation of hDPSCs. (a, b) Verification of the EVL overexpression efficiency in hDPSCs transfected with pM-EVL or pMV by western blot and qRT-PCR. (c) The expression of DSPP, DMP1, RUNX2, OCN, and OSX in hDPSCs transfected with pM-EVL and pMV by western blot on the 7th day. (d) The expression of DSPP, DMP1, ALP, RUNX2, OSX, OCN, and COL1A1 were analyzed in hDPSCs transfected with pM-EVL and pMV by qRT-PCR on the 3rd and 7th day. (e) ALP staining and quantitative intracellular ALP activity were determined in hDPSCs transfected with pM-EVL and pMV and cultured in GM or OM on the 3rd and 7th day. (f) Extracellular calcium deposition was visualized by alizarin red staining on the 21st day. Released dye in alizarin red staining was quantified by measuring absorbance at 562 nm. GAPDH was used as an internal control in qRT-PCR and western blot analyses. Data are shown as the mean ± SD (*n* = 3); ^∗^*P* ≤ 0.05, ^∗∗^*P* ≤ 0.01, ^∗∗∗^*P* ≤ 0.001.

Overexpression of EVL in hDPSCs was confirmed at both mRNA and protein expression levels (Figures 3(a) and 3(b)). qRT-PCR analysis showed that EVL overexpression led to a significant increase in DSPP, DMP1, ALP, RUNX2, OSX, OCN, and COL1A1 mRNA levels on days 3 and 7 (Figure 3(c)). Similarly, western blot analysis revealed that the protein levels of DSPP, DMP1, RUNX2, OSX, and OCN were significantly higher in the pM-EVL group than those in the control group (Figure 3(d)). Moreover, EVL facilitated osteo-/odontogenic differentiation of OM-cultured hDPSCs, as indicated by both ALP and ARS staining results, as well as ALP activity, but no difference was observed for GM-cultured hDPSCs (Figures 3(e) and 3(f)). Taken together, these results indicated that EVL promoted osteo-/odontogenic differentiation of hDPSCs in vitro.

### 3.5. Effect of EVL on MAPK Signaling Pathway during Differentiation of hDPSCs

**Figure 4:**
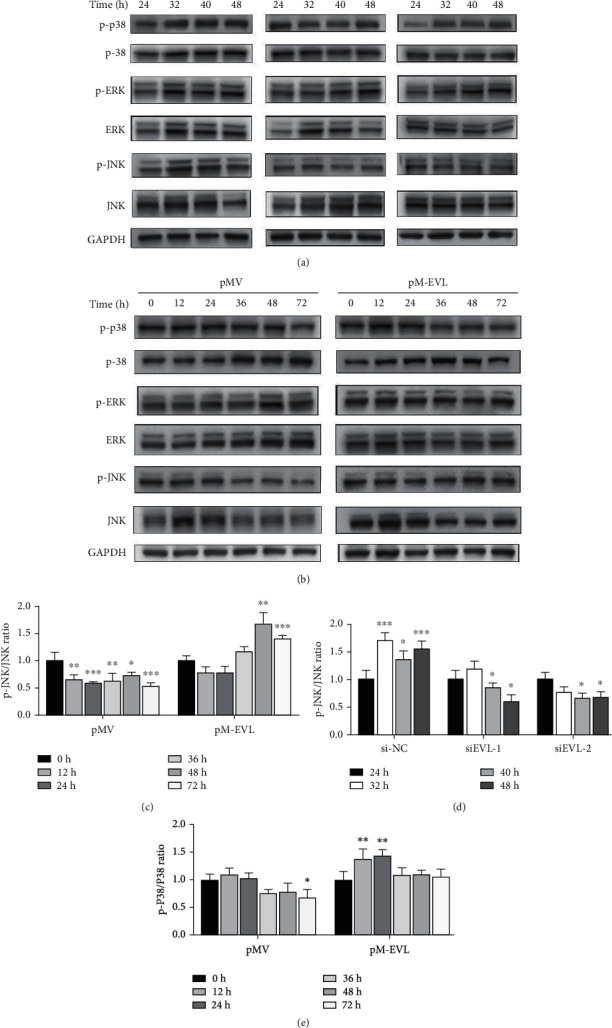
Involvement of the MAPK signaling pathway in EVL-mediated osteo-/odontogenic differentiation of hDPSCs. (a) Western blot analyses of p38 MAPK, p-p38 MAPK, ERK, p-ERK, JNK, and p-JNK expressions were measured in hDPSCs transfected with si-EVL and si-NC. (b) Protein expression levels of p38 MAPK, p-p38 MAPK, ERK, p-ERK, JNK, and p-JNK in hDPSCs transfected with pMV and pM-EVL. (c) The p-JNK/JNK ratio was measured in hDPSCs transfected with pMV and pM-EVL. (d) The p-JNK/JNK ratio was measured in hDPSCs transfected with si-EVL and si-NC. (e) The p-p38/p38 ratio was measured in hDPSCs transfected with pM-EVL and pMV. Data are shown as the mean ± SD (*n* = 3); ^∗^*P* ≤ 0.05, ^∗∗^*P* ≤ 0.01, ^∗∗∗^*P* ≤ 0.001.

Both gain- and loss-of-function assays demonstrated an important role of EVL in regulating the differentiation process of hDPSCs. Next, we sought to gain further insight into the molecular mechanisms underlying our findings. Recent studies have revealed a correlation between mitogen-activated protein kinase (MAPK) signaling pathway and EVL [38, 39]. Based on this finding, we conducted the following sets of experiments. First, we evaluated the expression levels of MAPK pathway-related proteins in si-NC-, si-EVL-, pMV-, and pM-EVL-transfected hDPSCs (Figures 4(a) and 4(b)). Western blot results showed that the p-JNK/JNK ratio was significantly elevated in the pM-EVL group compared with the pMV group (Figure 4(c)), and silencing of EVL decreased this ratio in the si-EVL group compared with the control group (Figure 4(d)), and there were no significant differences in protein level of JNK among these groups. Similarly, the p-p38/p38 ratio was increased in the pM-EVL group compared with the pMV group (Figure 4(e)), but no difference in p-p38/p38 ratio was observed between the si-EVL and negative control group. No significant differences were observed in protein level of ERK, p38, and p-ERK/ERK ratio among groups. These results indicated that EVL activated JNK signaling pathway during osteo-/odontogenic differentiation of hDPSCs.

### 3.6. EVL Regulated the Differentiation of hDPSCs through JNK Signaling Pathway

**Figure 5:**
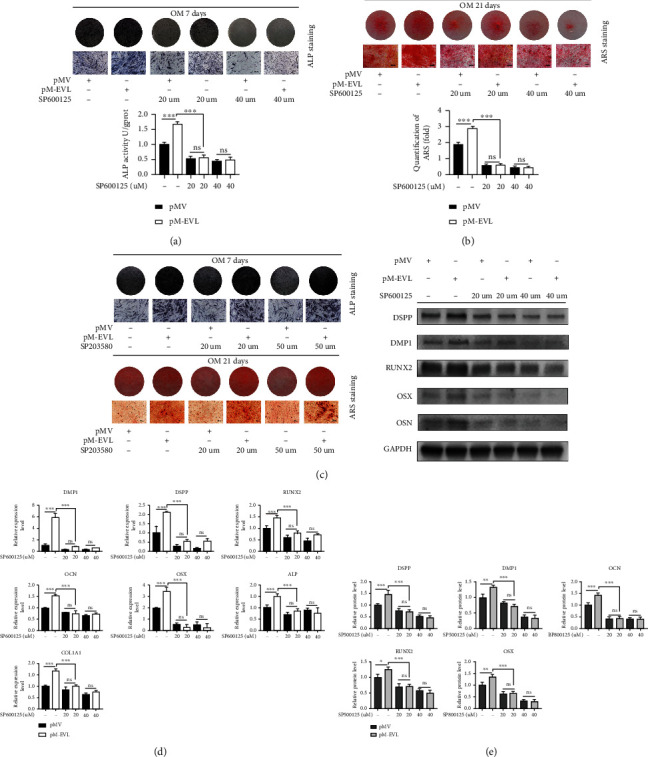
EVL enhanced hDPSCs osteo-/odontogenic differentiation via activating JNK signaling. (a) Pretreatment with 20 or 40 *μ*M SP600125 for 1 h, ALP staining and quantitative intracellular ALP activity were determined in hDPSCs transfected with pM-EVL on the 7th day. (b) Extracellular calcium deposition was visualized by alizarin red staining on the 21st day. Released dye in alizarin red staining was quantified by measuring absorbance at 562 nm. (c) Pretreatment with 20 or 50 *μ*M SB203580 for 1 h, ALP and ARS staining were determined in hDPSCs transfected with pM-EVL. (d) Pretreatment with 20 or 40 *μ*M SP600125 for 1 h, the expression of DSPP, DMP1, ALP, RUNX2, OSX, OCN, and COL1A1 is analyzed in hDPSCs transfected with pM-EVL by qRT-PCR on the 7th day. (e) The expression of DSPP, DMP1, RUNX2, OCN, and OSX in hDPSCs transfected with pM-EVL is analyzed by western blot on the 7th day. GAPDH was used as an internal control in qRT-PCR and western blot analyses. Data are shown as the mean ± SD (*n* = 3); ^∗^*P* ≤ 0.05, ^∗∗^*P* ≤ 0.01, ^∗∗∗^*P* ≤ 0.001.

Next, we aimed to assess whether the JNK pathway contributed to the osteogenesis-/odontogenesis-promoting effects of EVL. SP600125, a specific inhibitor of JNK pathway, was used to verify the involvement of JNK pathway in EVL-mediated osteo-/odontogenic differentiation of hDPSCs. We used SP600125 at a concentration of 20 *μ*M and 40 *μ*M to suppress the activity of JNK for one hour before pMV and pM-EVL transfection. Both ALP and ARS staining, as well as ALP activity assay, revealed that SP600125 attenuated the promoting effects of EVL on osteo-/odontogenic differentiation (Figures 5(a) and 5(b)). In this study, we noted that EVL overexpression increased phosphorylation of p38 MAPK. To further explore the involvement of p38 MAPK during the EVL-induced differentiation of hDPSCs, specific pathway inhibitor SB203580 was applied for blocking the p38 MAPK pathway. Notably, the ALP and ARS staining, which were enhanced by EVL, were not suppressed in EVL+ SB203580 groups (Figure 5(c)), suggesting that p38 MAPK signaling pathway was not involved in EVL-mediated differentiation of hDPSCs. Furthermore, pretreatment of hDPSCs with SP600125 resulted in a downregulated expression of key osteo-/odontogenic markers at both mRNA and protein levels, in comparison with the pM-EVL transfection controls (Figures 5(d) and 5(e)). These results suggested that EVL promoted hDPSCs differentiation mainly via the JNK pathway.

## 4. Discussion

hDPSCs are a promising cell source in bone and teeth tissue engineering Thus, it is necessary to figure out the molecular mechanisms underlying hDPSCs osteo-/odontogenic differentiation, which may provide us with a better understanding of the biological properties and therapeutic potential of hDPSCs.

Osteo-/odontogenic differentiation of hDPSCs is a complex process which is regulated by multiple factors. The reorganization of the actin cytoskeleton plays a crucial role during stem cells differentiation [22, 40, 41]. A large number of studies have shown that there is an inverse relationship between actin polymerization and adipogenesis, whereas there is a direct correlation between actin polymerization and osteogenesis [27, 28, 42–44]. The dynamic nature of the actin cytoskeleton is determined by the actions of numerous actin-binding proteins, such as cofilin, profilin, CapZ, Arp2/3, and Ena/VASP family [45–47]. EVL, one of the Ena/VASP family involved in actin assembly and elongation, has been implicated in several types of cells with cell proliferation and differentiation by enhancing actin polymerization [37, 39, 48–50]. However, the effect of EVL on osteo-/odontogenic differentiation of hDPSCs still remains unknown.

In the present study, we investigated for the first time the functional roles of EVL in hDPSC differentiation and the underlying molecular mechanisms. The results indicated that the mRNA and protein expression levels of EVL were significantly increased during the differentiation of hDPSCs. It is notable that EVL expression trend downward at day 10 of osteogenic differentiation, suggesting that EVL may play a regulatory role mainly in the early stage of osteogenesis. To further investigate whether EVL could promote hDPSC differentiation in vitro, we examined the expressions of key osteo-/odontogenic markers using gain- and loss-of-function strategies. The results revealed that overexpression of EVL promoted the mRNA and protein expressions of key osteo-/odontogenic markers, such as DSPP, DMP1, RUNX2, ALP, OSX, OCN, and COL1A1. Moreover, ALP and ARS staining, and ALP activity were increased in the pM-EVL group, whereas downregulation of EVL exerted the opposite effects.

Next, we sought to investigate the molecular mechanisms by which EVL regulated the differentiation of hDPSCs. It is well known that the MAPK signaling pathway regulates a variety of biological processes, including development, proliferation, differentiation, motility, survival, and apoptosis [51, 52] .Some studies showed that MAPK signaling pathway played a crucial role in mediating osteo-/odontogenic differentiation and are likely regulated by EVL [38, 39, 53–55]. In the present study, to investigate whether EVL enhanced osteo-/odontogenic differentiation through activating MAPK pathway, we examined the activation of key signaling molecules of the MAPK pathway, including p38 MAPKs, JNKs, and ERKs of MAPK pathway in cells transfected with pM-EVL, si-EVL, and negative control. The results showed that EVL overexpression significantly enhanced JNK and p38 phosphorylation levels, and EVL silencing resulted in a reduced JNK phosphorylation level. Providing new evidence for the possible involvement of JNK and p38 MAPK signaling in EVL-enhanced differentiation of hDPSCs. Furthermore, both JNK and p38 MAPK signal pathway inhibitors were used to confirm which signal pathway might play a key role in EVL-enhanced differentiation of hDPSCs. After blocking the signal pathways, the JNK inhibitor attenuated the promoting effect of EVL on hDPSCs osteo-/odontogenic differentiation, which was characterized by reduced ALP activity, calcium deposition, and expressions of key osteo-/odontogenic markers. However, p38 MAPK inhibitor failed to attenuate EVL-enhanced osteo-/odontogenic differentiation, indicating that p38 MAPK pathway does not participate in EVL-enhanced osteo-/odontogenic differentiation in hDPSCs. These data demonstrate that JNK pathway activation was the primary mechanism underlying EVL-enhanced osteo-/odontogenic differentiation of hDPSCs, but further in-depth research is necessary to explore the specific mechanisms in this process.

Pulp necrosis of immature permanent teeth from pulpitis, periapical periodontitis, or trauma halt further root development and compromise the longevity of the teeth [56, 57]. Current approaches for treating immature permanent teeth with pulpal necrosis do not reliably achieve continued root development and restore functional competence of the pulp tissue [58, 59]. Recently, stem cell-based techniques have shown immense potential for regeneration of the dentin-pulp complex [6, 7, 60]. Within this context, the ability of EVL to enhance the osteo-/odontogenic commitment in hDPSCs may prove rewarding in the promotion of root development and reinforcement of dentinal walls by deposition of hard tissue. In the present study, we confirmed the vital role of EVL in modulating osteo-/odontogenic differentiation of hDPSCs, and clarified the involvement of JNK signaling pathway in EVL-induced osteo-/odontogenic differentiation, providing a better understanding of the molecular mechanisms that drove hDPSCs differentiation, and potentially introducing a new method to the field of dental pulp tissue engineering and endodontic therapy. Further studies should be pursued to explore the effect of EVL-mediated regeneration in animal models.

In conclusion, we showed for the first time the role of EVL in modulating the osteo/odontoblastic differentiation of hDPSCs. These observations set the basis for using EVL as a potential and alternative tool for the differentiation ability of human stem cells, favoring their use in a therapeutic approach.

## 5. Conclusions

In conclusion, this study demonstrated that EVL plays a key role in promoting osteo-/odontogenic differentiation of hDPSCs via activating JNK pathway, providing important insight into the mechanisms underlying functional regulation of hDPSCs, and indicating that EVL has a significant role in maintaining the osteo-/odontogenic differentiation potential of hDPSCs.

## Figures and Tables

**Table 1:** RT-PCR primers for the target genes.

Target gene	Primer sequence forward(5′ to 3′)	Primer sequence reverse(5′ to 3′)
GAPDH	CGGGAAGCTTGTCATCAATGG	GGCAGTGATGGCATGGACTG
RUNX2	TGGTTACTGTCATGGCGGGTA	TCTCAGATCGTTGAACCTTGCTA
DMP-1	CTCCGAGTTGGACGATGAGG	CATGCCTGCACTGTTCATTC
DSPP	ATATTGAGGGCTGGAATGGGGA	TTTGTGGCTCCAGCATTGTCA
OSX	CCTCCTCAGCTCACCTTCTC	GTTGGGAGCCCAAATAGAAA
ALP	GACCTCCTCGGAAGACACTC	TGAAGGGCTTCTTGTCTGTG
OCN	AGCAAAGGTGCAGCCTTTGT	GCGCCTGGGTCTCTTCACT
COL1A1	GAGGGCCAAGACGAAGACATC	CAGATCACGTCATCGCACAAC
EVL	TGCTGCTCCATCACTTGTCT	CTCCAATGCAATGCTGTTTG

**Table 2:** The target sequences of the siRNAs.

siRNA	Target sequence
si-NC	TTCTCCGAACGTGTCACGT
si-EVL-1	CATCATGAATTCCCAAGAA
si-EVL-2	GCTTAAACTTTGCAAGTAA

## Data Availability

The data used to support the findings of this study are available from the corresponding author upon request.
